# Identification of novel PTPRQ phosphatase inhibitors based on the virtual screening with docking simulations

**DOI:** 10.1186/1742-4682-10-49

**Published:** 2013-08-28

**Authors:** Hwangseo Park, Keum Ran Yu, Bonsu Ku, Bo Yeon Kim, Seung Jun Kim

**Affiliations:** 1 Department of Bioscience and Biotechnology, Sejong University, 98 Kunja-Dong, Kwangjin-Ku, Seoul 143-747, Korea; 2 Medical Proteomics Research Center, Korea Research Institute of Bioscience and Medical Biotechnology, 125 Gwahak-ro, Yuseong-gu, Daejeon 305-806, Korea; 3 Chemical Biology Research Center, Korea Research Institute of Bioscience and Biotechnology, 125 Gwahak-ro, Yuseong-gu, Daejeon 305-806, Korea

**Keywords:** Virtual screening, PTPRQ, Inhibitor, Docking, Antiobestic agents

## Abstract

Protein tyrosine phosphatase receptor type Q (PTPRQ) is an unusual PTP that has intrinsic dephosphorylating activity for various phosphatidyl inositides instead of phospho-tyrosine substrates. Although PTPRQ was known to be involved in the pathogenesis of obesity, no small-molecule inhibitor has been reported so far. Here we report six novel PTPRQ inhibitors identified with computer-aided drug design protocol involving the virtual screening with docking simulations and enzyme inhibition assay. These inhibitors exhibit moderate potencies against PTPRQ with the associated IC_50_ values ranging from 29 to 86 μM. Because the newly discovered inhibitors were also computationally screened for having desirable physicochemical properties as a drug candidate, they deserve consideration for further development by structure-activity relationship studies to optimize the antiobestic activities. Structural features relevant to the stabilization of the inhibitors in the active site of PTPRQ are addressed in detail.

## Introduction

Protein tyrosine phosphatases (PTPs) catalyze the hydrolysis of the phosphorylated tyrosine residues of protein substrates, which is a hallmark of cellular signal transduction. Because these dephosphorylation activities of PTPs have been implicated in a variety of cellular processes, abnormal PTP activities may cause various diseases including cancer, diabetes, and immune deficiencies [1]. Total 38 members of PTP family (21 receptor-type PTPs and 17 nonreceptor-type PTPs) are known to have specificity for the phosphorylated tyrosine substrates [2]. Most PTPs share a highly conserved catalytic module that plays a crucial role in the enzymatic action for dephosphorylation reaction. This catalytic core comprises a PTP loop (Cys-Ser-Xaa-Gly-Xaa-Gly-Arg-Thr/Ser), WPD loop, and Q loop [3]. The invariant cysteine is located at the bottom of the PTP loop to act as a nucleophile to attack the substrate phosphorous atom, while the side-chain guanidinium ion of the conserved Arg residue in the PTP loop stabilizes the negative charge on the oxygen atoms of the substrate accumulated during the hydrolysis reaction. The conserved Gln and Asp residues in the Q and WPD loops also participate in the hydrolysis reaction of the substrate through the role of a general acid/base catalyst [3].

PTP receptor type Q (PTPRQ) is a member of the receptor type PTP family that contains 18 extracellular fibronectin domains and one cytoplasmic catalytic domain. Although the primary sequence of the catalytic domain of PTPRQ (PTPRQ-C) shows a high degree of similarity to those of the known PTPs, PTPRQ displays an unusual catalytic behavior. For example, it has intrinsic dephosphorylating activity for various phosphatidyl inositides (PIs) but not for phospho-tyrosine substrates [4]. Furthermore, PTPRQ negatively regulates the proliferation and survival of cells by lowering the level of phosphoinositol phosphates (PIPs) [5]. This characteristic dephosphorylating activity can be attributed in a large part to the difference in amino-acid sequence of the WPD loop in which the conserved aspartate is replaced with a glutamate. This hypothesis was supported by the experimental finding that the reverse mutation of glutamate to aspartate in the WPE motif caused PTPRQ to gain catalytic activity toward pTyr while losing the activity with respect to PI substrates [5]. Four catalytically active members of the classical PTP family (PTPRQ, PTPRU, PTPD1 and HDPTP) in human genome possess the WPE motif instead of the WPD one, the structures of which have not characterized yet except for PTPRQ. PTPRQ is also homologous to a few PTPs such as phosphatase and tensin homolog (PTEN) and myotubularin phosphatases, which have the catalytic capability to dephosphorylate PIs [2]. A line of experimental evidence showed that the loss of *PTPRQ* gene could lead to the hearing impairment associated with vestibular dysfunction [6-8]. It was also demonstrated that the overexpression of PTPRQ caused the differentiation of mesenchymal stem cells (MSCs) into adipocytes, which leads to the pathogenesis of obesity [9]. This indicates that PTPRQ can serve as an effective target for development of new antiobestic drugs.

Very recently, X-ray crystal structure of human PTPRQ has been reported in complex with the sulfate ion bound in the active site as a surrogate for the phosphate group of substrates [10]. In this structure, PTPRQ adopts an open conformation in which the residues of WPE loop stay distant from the active site. It has a flatter active site than other PTPs to accommodate the PIP substrates that are larger than the phosphorylated tyrosine. The presence of structural information about the nature of the interactions between PTPRQ and small-molecule ligands can make it a plausible task to design the potent inhibitors that may develop into an antiobestic drug. Nonetheless, the discovery of PTPRQ inhibitors has lagged behind the biological and structural studies. To the best of our knowledge, no small-molecule PTPRQ inhibitor has been reported so far in the literature at least. In this paper, we report the novel classes of PTPRQ inhibitors identified through the structure-based drug design protocol involving the virtual screening with docking simulations and *in vitro* enzyme assay. Computer-aided drug design has not always been successful due to the inaccuracy in the scoring function, which leads to a weak correlation between the computational predictions and experimental results for binding affinities [11]. Therefore, we implement an accurate solvation free energy function into the scoring function to enhance the accuracy in calculating the binding free energies between PTPRQ and the putative inhibitors. This modification of the scoring function seems to improve the potential for designing the new inhibitors with high activity [12]. It will be shown that docking simulations with the improved binding free energy function can be a useful tool for enriching the chemical library with molecules that are likely to have desired biological activities, as well as for elucidating the activities of the identified inhibitors.

## Methods

3D atomic coordinates in the X-ray crystal structure of human PTPRQ in complex with the sulfate ion as a substrate analogue (PDB code: 4ikc) were selected as the receptor model in the virtual screening. After removing the crystallographic water molecules, hydrogen atoms were added to each protein atom. A special attention was paid to assign the protonation states of the ionizable Asp, Glu, His, and Lys residues in the original X-ray structure of PTPRQ. The side chains of Asp and Glu residues were assumed to be neutral if one of their carboxylate oxygens pointed toward a hydrogen-bond accepting group including the backbone aminocarbonyl oxygen at a distance within 3.5 Å, a generally accepted distance limit for a hydrogen bond of moderate strength [13]. Similarly, the lysine side chains were assumed to be protonated unless the NZ atom was in proximity of a hydrogen-bond donating group. The same procedure was also applied to determine the protonation states of ND and NE atoms in His residues.

The docking library for PTPRQ comprising about 260,000 synthetic and natural compounds was constructed from the latest version of the chemical database distributed by Interbioscreen (http://www.ibscreen.com) containing approximately 500,000 synthetic and natural compounds. Prior to the virtual screening with docking simulations, they were filtrated on the basis of Lipinski’s “Rule of Five” to adopt only the compounds with the physicochemical properties of potential drug candidates [14] and without reactive functional group(s). To remove the structural redundancies in the chemical library, structurally similar compounds with a Tanimoto coefficient exceeding 0.85 were clustered into a single representative molecule. Molecular similarities were measured using the fingerprints of each molecule, generated using the Daylight software as an ASCII string of 1’s and 0’s. In this way, a docking library consisting of 260,000 compounds was constructed. All compounds included in the docking library were then processed with the CORINA program to generate their 3D atomic coordinates, followed by the assignment of Gasteiger-Marsilli atomic charges [15]. We used the AutoDock program [16] in the virtual screening of PTPRQ inhibitors because the outperformance of its scoring function over those of the others had been shown in several target proteins [17]. AMBER force field parameters were assigned for calculating the van der Waals interactions and the internal energy of a ligand as implemented in the original AutoDock program. Docking simulations with AutoDock were then carried out in the active site of PTPRQ to score and rank the compounds in the docking library according to their calculated binding affinities.

In the actual docking simulation of the compounds in the docking library, we used the empirical AutoDock scoring function improved by the implementation of a new solvation model for a compound. The modified scoring function can be expressed in the following form.

(1) $$\begin{array}{l} \Delta G_{\mathrm{bind}}^{\mathrm{aq}}=W_{\mathrm{vdW}}\sum_{i=1}\sum_{j=1}\left(\frac{A_{\mathrm{ij}}}{r_{\mathrm{ij}}^{12}}-\frac{B_{\mathrm{ij}}}{r_{\mathrm{ij}}^{6}}\right)+W_{\mathrm{hbond}}\sum_{i=1}\sum_{j=1}E\left(t\right)\left(\frac{C_{\mathrm{ij}}}{r_{\mathrm{ij}}^{12}}-\frac{D_{\mathrm{ij}}}{r_{\mathrm{ij}}^{10}}\right)+W_{\mathrm{elec}}\sum_{i=1}\sum_{j=1}\frac{q_{i}q_{j}}{\epsilon \left(r_{\mathrm{ij}}\right)r_{\mathrm{ij}}}\;\\ \;+W_{\mathrm{tor}}N_{\mathrm{tor}}+W_{\mathrm{sol}}\sum_{i=1}\left\{S_{i}O_{i}^{\max}+\left(P_{i}-S_{i}\right)\sum_{j\neq i}V_{j}e^{-\frac{r_{\mathrm{ij}}^{2}}{2\sigma^{2}}}\right\} \end{array}$$

Here *W*_*vdW*_, *W*_*hbond*_, *W*_*elec*_, *W*_*tor*_, and *W*_*sol*_ are the weighting factors of van der Waals, hydrogen bond, electrostatic interactions, torsional term, and desolvation energy of the inhibitors, respectively. *r*_*ij*_ represents the interatomic distance, and *A*_*ij*_, *B*_*ij*_, *C*_*ij*_, and *D*_*ij*_ are related to the depths of the potential energy well and the equilibrium separations between the two atoms. The hydrogen bond term has an additional weighting factor, *E*(*t*), representing the angle-dependent directionality. Cubic equation approach was applied to obtain the dielectric constant required in computing the interatomic electrostatic interactions between PTPRQ and a ligand molecule [18]. In the entropic term, *N*_*tor*_ is the number of rotatable bonds in the ligand. In the desolvation term, *S*_*i*_ and *V*_*i*_ are the solvation parameter and the fragmental volume of atom *i*[19], respectively, while *O*_*i*_^max^ stands for the maximum atomic occupancy. The self-solvation parameter *P*_*i*_ represents the extent of the stabilization of the solute atom *i* due to the intramolecular interactions with the rest of solute atoms. Inclusion of this self-solvation effect in the scoring function is necessary because the calculated molecular solvation free energies were shown to be inaccurate in the absence of the self-solvation term [20]. To calculate the contribution of molecular solvation free energy term in Eq. (1), we used the atomic parameters developed by Choi and coworkers [20]. This modification of the solvation free energy term is expected to increase the accuracy in virtual screening because the underestimation of ligand solvation often leads to the overestimation of the binding affinity of a ligand with many polar atoms [12].

The catalytic domain of PTPRQ (PTPRQ-C, residues 2661–2948) was subcloned into pET28a and overexpressed using *Escherichia coli* BL21 (DE3) strain. Cells were grown at 291 K after induction with 0.1 mM IPTG for 20 hours. His-tagged PTPRQ-C was purified by nickel-affinity chromatography. 150 compounds selected from the precedent virtual screening were evaluated for their in vitro inhibitory activity against the recombinant human PTPRQ. Initial inhibitor screening was performed by monitoring the extent of hydrolysis of *p*-Nitrophenyl Phosphate (pNPP) with a spectrofluorometric assay. The purified PTPRQ-C (1.5 μM), pNPP (5 mM), and a candidate inhibitor were incubated in the reaction mixture containing 50 mM Bis-Tris (pH 6.0), 2 mM dithiothreitol for 60 minutes. This enzymatic reaction was stopped with the addition of sodium hydroxide (0.5 M). The phosphatase activities were then checked by the absorbance changes due to the hydrolysis of the substrate at 405 nm. IC_50_ values of the inhibitors were determined from direct regression curve analysis.

Six PTPRQ inhibitors identified under the above reaction conditions were further investigated using PI(3,4,5)P_3_ as the substrate (Cayman Chemical). The enzymatic activity of PTPRQ was measured in 80 μL reaction mixture containing 50 mM Tris–HCl (pH 6.0), 10 mM dithiothreitol, 300 μM PI(3,4,5)P_3_, 100 μM inhibitor, and 1.5 μM of the purified catalytic domain of PTPRQ. The mixture was incubated for 60 minutes at 310 K, and the enzymatic reaction was stopped by the addition of 20 μL of malachite green/ammonium molybdate reagent (Bioassay systems). The absorbance was measured at 650 nm using a plate reader.

## Results and discussion

**Figure 1:**
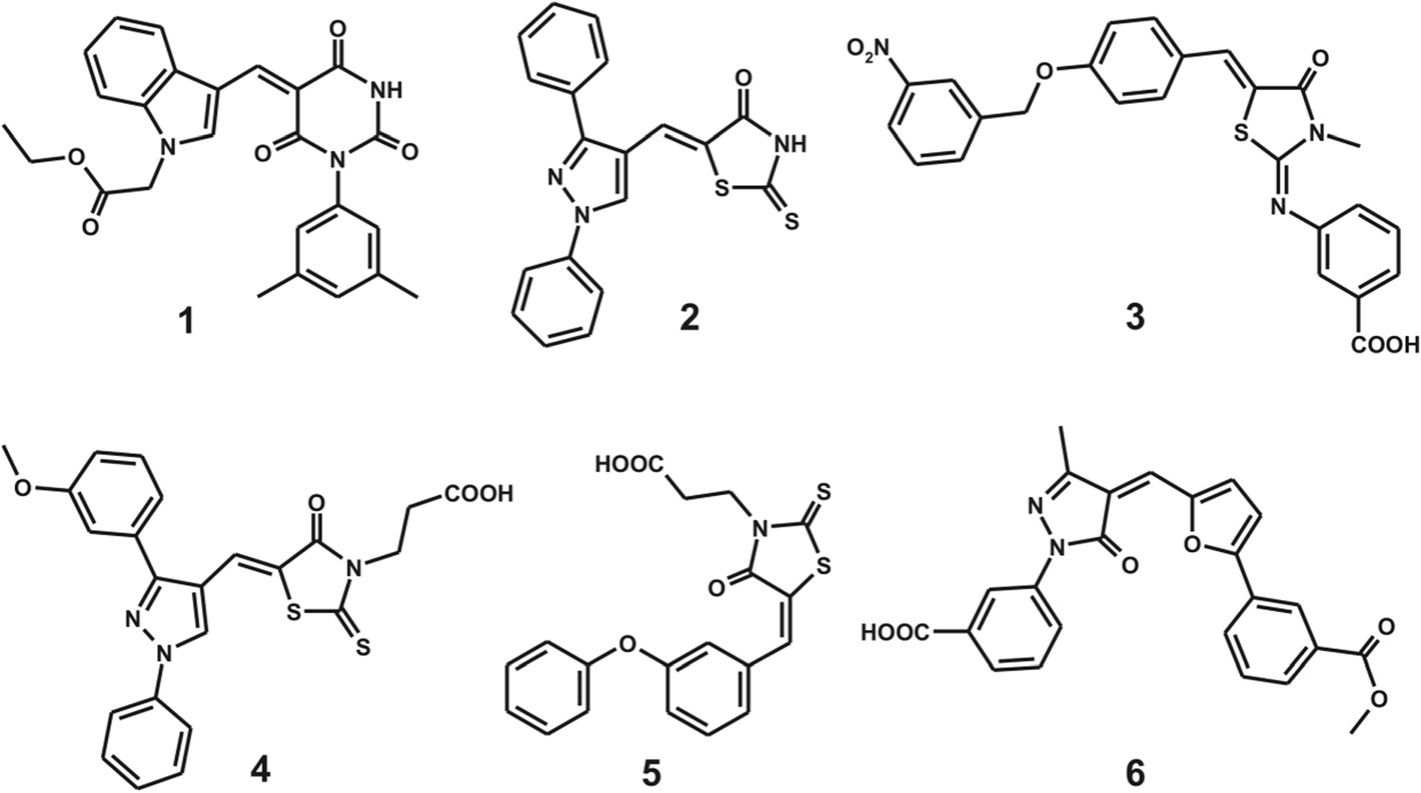
Chemical structures of the PTPRQ inhibitors identified from virtual screening.

Of the 260,000 compounds screened with docking simulations in the active site of PTPRQ, 150 top-scored compounds were selected as virtual hits. All of them were available from the compound supplier and were tested for having the inhibitory activity against PTPRQ at the concentration of 100 μM. As a result, we identified the six compounds that inhibited the catalytic activity of PTPRQ by more than 50% at 100 μM, which were selected to determine the IC_50_ values. The chemical structures and the inhibitory activities of the identified PTPRQ inhibitors are shown in Figure 1 and Table 1, respectively. The structures of remaining 144 inactive compunds are also provided in Additional file 1. We note that **1**, **2**, and **3**–**6** include pyrimidine-2,4,6-trione, 2-imino-thiazolidin-4-one, and carboxylate moieties in the molecular structures, respectively. These chemical groups are expected to serve as a surrogate for the substrate phosphate group with negative charge. To the best of our knowledge, compounds **1**–**6** have not been reported as a phosphatase inhibitor so far. In addition, no additional biological activity was found for the six inhibitors at least in the two most popular chemical databases, ChEMBL and PubChem.

**Table 1:** **IC**_**50 **_**values (in μM) of 1–6 against PTPRQ**

**Compounds**	**IC**_**50**_
**1**	29.9
**2**	85.7
**3**	63.8
**4**	44.8
**5**	65.3
**6**	73.1

As can be seen in Table 1, the potencies of the six PTPRQ inhibitors are moderate with the IC_50_ values ranging from 29.9 to 85.7 μM. These modest inhibitory activities can be understood because PTPRQ has a flat and shallow active site, which makes it difficult for the inhibitors to be fully accommodated [21]. To improve the inhibitory activities, therefore, some chemical groups should be added to **1**–**6** in such a way that the resulting derivatives can be stabilized not only in the active site but also in other peripheral binding pockets. Despite the modest inhibitor potencies, **1**–**6** deserve consideration for further development by structure-activity relationship (SAR) studies to optimize the antiobestic activities because they are structurally diverse and were computationally screened for having desirable physicochemical properties as a drug candidate.

**Figure 2:**
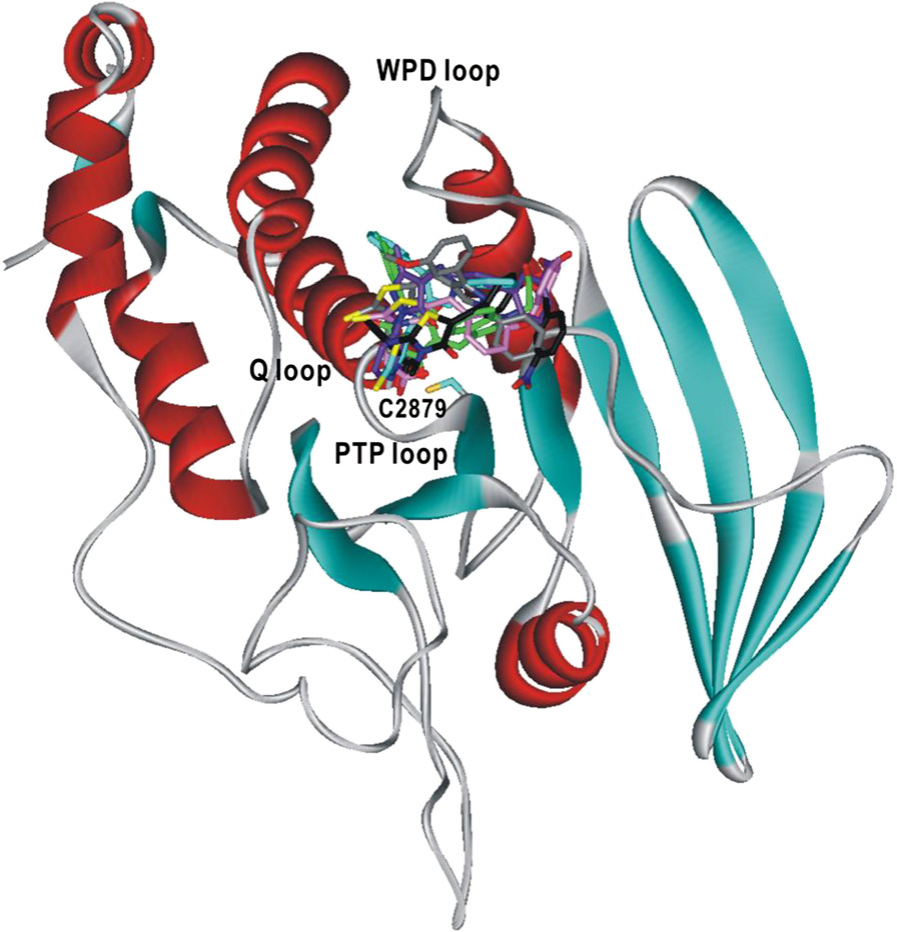
**Comparative view of the binding modes of the PTPRQ inhibitors.** Carbon atoms of **1**–**6** are indicated in green, cyan, black, gray, pink, and violet, respectively.

To obtain structural insight into the inhibitory mechanisms of the identified PTPRQ inhibitors, their binding modes in the active site were investigated in a comparative fashion. Figure 2 shows the lowest-energy conformations of **1**–**6** in the active site gorge of PTPRQ calculated with the modified AutoDock program. The results of these docking simulations are self-consistent in the sense that the functional groups of similar chemical character are placed in similar ways with comparable interactions with the protein groups. As revealed by the superimposed structures of **1**–**6** in Figure 2, for example, the polar groups (pyrimidine-2,4,6-trione, 2-imino-thiazolidin-4-one, and carboxylate moieties) point toward the catalytic cysteine residue (Cys2879) located at the bottom of active site while the hydrophobic groups are directed to the WPD loop that resides above the active site. These common features in the calculated binding modes indicate that PTPRQ inhibitors should include an effective surrogate for the substrate phosphate group and simultaneously the hydrophobic groups for binding to the WPD loop. In order to examine the possibility of the allosteric inhibition of PTPRQ by **1**–**6**, docking simulations were carried out with the grid maps for the receptor model so as to include the entire part of the catalytic domain of PTPRQ. However, the binding configuration in which an inhibitor resides outside the active site was not observed for any of the new inhibitors. These results support the possibility that the inhibitors would impair the catalytic activity of PTPRQ through the specific binding in the active site.

**Figure 3:**
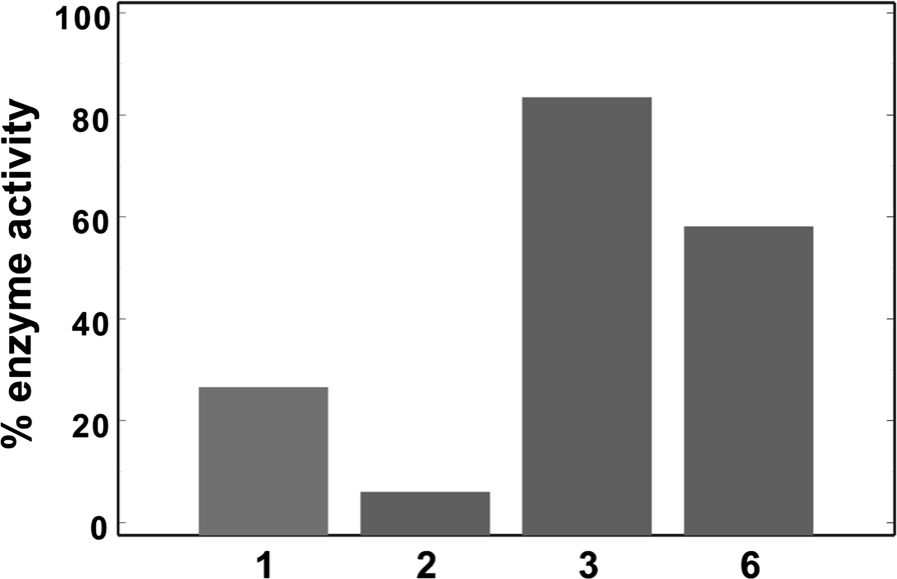
**The inhibitory activity of 1, 2, 3, and 6 against the hydrolysis of PI(3,4,5)P**_**3 **_**by PTPRQ.** The enzymatic activity was assayed by monitoring the amount of released phosphate ion using malachite green reagents.

Because PTPRQ functions on PIPs rather than the phosphorylated tyrosine, we also examined whether **1**–**6** could inhibit the dephosphorylation activity of PTPRQ with respect to the PI(3,4,5)P_3_ substrate. Under the enzymatic reaction condition including 1.5 μM PTPRQ-C, 300 μM PI(3,4,5)P_3_, and 100 μM inhibitor, **1** and **2** exhibit a significant inhibitory activity against the catalytic capability of PTPRQ (Figure 3). Treatments of **3** and **6** in the reaction mixture have also an effect of reducing PTPRQ activity although the extents of inhibition decrease significantly when compared to those of **1** and **2**. These results confirm that **1**, **2**, **3**, and **6** are capable of impairing the enzymatic activity of PTPRQ. However, the inhibitory activities **4** and **5** with respect to the catalytic hydrolysis of PI(3,4,5)P_3_ by PTPRQ could not be measured due to their nonspecific color reactions with the malachite green used for the detection of dephosphorylation. The development of a new assay method is underway so that the inhibitory activities of all putative inhibitors with respect to the lipid dephosphorylation of PTPRQ can be measured successfully.

**Figure 4:**
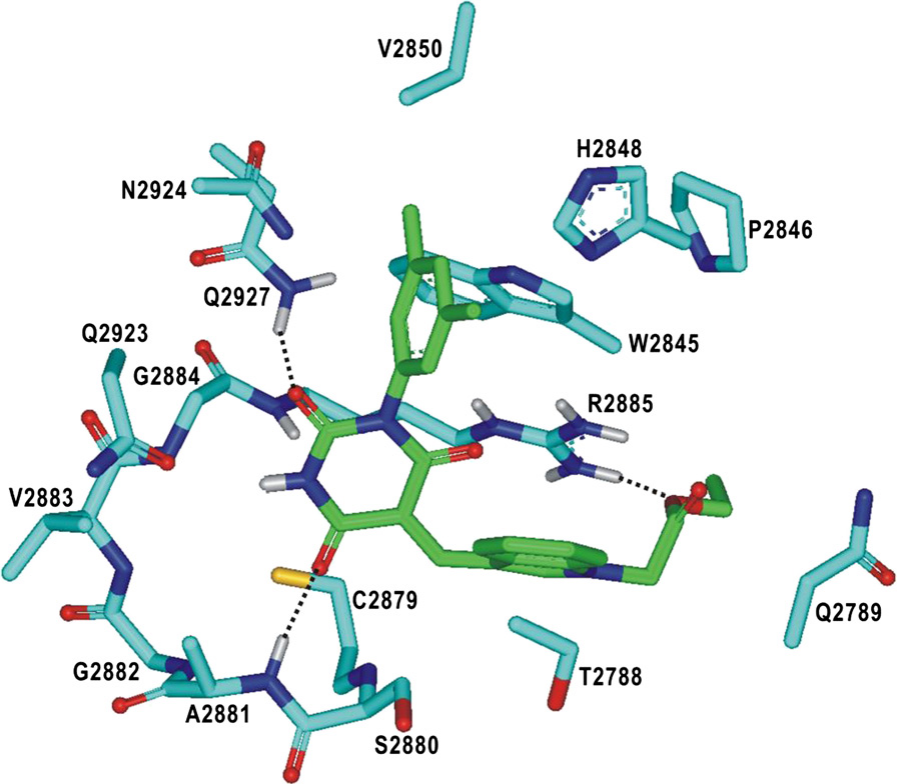
**Calculated binding mode of 1 in the active site of PTPRQ.** Carbon atoms of the protein and the ligand are indicated in cyan and green, respectively. Each dotted line indicates a hydrogen bond.

We now turn to the identification of the detailed interactions responsible for the stabilization of PTPRQ inhibitors in the active site. The calculated binding mode of **1** in the active site is shown in Figure 4. The inhibitor appears to be in a close contact with Cys2879–Arg2885, Trp2845–Val2850, and Gln2923–Gln2927, which belong to the PTP, WPD, and Q loops, respectively. We note that the two aminocarbonyl oxygen atoms of **1** receive the hydrogen bonds from the backbone amidic nitrogen of Ala2881 and the side-chain amidic nitrogen of Gln2927. These two hydrogen bonds seem to play a role of anchor in positioning the inhibitor in the active site. It is also noteworthy that the two aminocarbonyl oxygens of **1** reside in the vicinity of the side-chain thiolate group of Cys2879 with the associated interatomic distances within 4.0 Å. Judging from the proximity to Cys2879 and the formation of the multiple hydrogen bonds in the active site, the pyrimidine-2,4,6-trione moiety of **1** is likely to serve as an effective surrogate for the substrate phosphate group. A stable hydrogen bond is also established between the terminal ester group of **1** and the side-chain guanidinium ion of Arg2885. The inhibitor **1** can be further stabilized in the active site of PTPRQ by van der Waals interactions of its nonpolor groups with the hydrophobic side chains of Trp2845, Pro2846, His2848, and Val2850. Thus, the overall structural features derived from docking simulations indicate that the inhibitory activity of **1** stems from the multiple hydrogen bonds and hydrophobic interactions established simultaneously in the active site.

**Figure 5:**
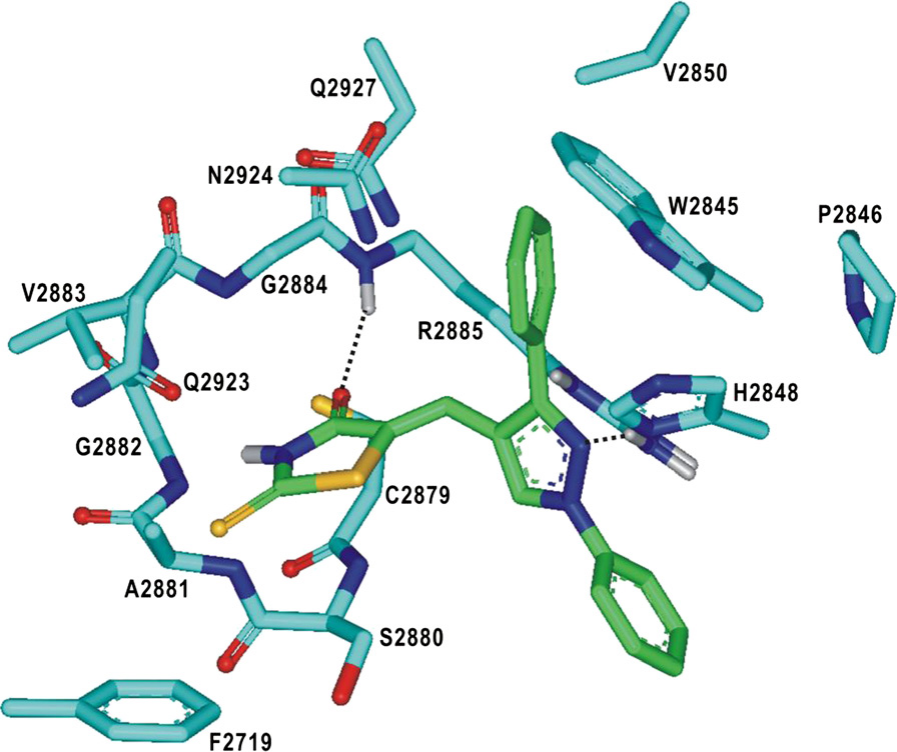
**Calculated binding mode of 2 in the active site of PTPRQ.** Carbon atoms of the protein and the ligand are indicated in cyan and green, respectively. Each dotted line indicates a hydrogen bond.

Figure 5 shows the lowest-energy binding mode of **2** in the active site of PTPRQ. The binding mode of **2** differs from that of **1** in that the role of hydrogen bond donor with respect to the inhibitor aminocarbonyl oxygen is played by the backbone amidic groups of Arg2885 instead of Ala2881 and Gln2927. An additional hydrogen bond appears to be established between the side-chain guanidinium ion of Arg2885 and the pyrazole ring of **2**, which should also be a significant binding force to stabilize the inhibitor in the active site. It is noteworthy that the number of hydrogen bonds decreases from three in the PTPRQ-**1** to two in the PTPRQ-**2** complex, which would have an effect of lowering the inhibitory activity. Hydrophobic interactions in the PTPRQ-**2** complex are established in similar way to those in the PTPRQ-**1** complex: two terminal phenyl rings of **2** form the van der Waals contacts with the side chains of Trp2845, Pro2846, His2848, and Val2850. Therefore, the relatively lower inhibitory activity of **2** than **1** can be attributed to the loss of one hydrogen bond. Binding modes of **3**–**6** appear to be similar to those of **1** and **2** in that the terminal carboxylate and the aromatic groups are stabilized by the hydrogen bonds with the amino-acid residues around the PTP loop and the hydrophobic interactions with nonpolar residues on the WPD loop, respectively (data not shown here). It is thus found to be a common feature in binding modes of **1**–**6** that multiple hydrogen bonds and hydrophobic interactions contribute to the stabilization of the inhibitors in the active site of PTPRQ in a cooperative fashion.

Because the selectivity has been one of the most important issues in the development of phosphatase inhibitors, we compared the inhibitory activities of **1**–**6** for PTPRQ to those for its homologous protein, PTP receptor type O (PTPRO). These inhibition assays for selectivity were done in duplicates at the inhibitor concentration of 100 μM. As can be seen in Table 2, all six compounds have a significant inhibitory activity against both PTPRQ and PTPRO, which exemplifies the difficulty in the discovery of specific inhibitors. The simultaneous inhibitions of PTPRQ and PTPRO by **1**–**6** are actually not surprising because they share a highly conserved catalytic module. To obtain the specific inhibitors for PTPRQ, therefore, it seems that some chemical groups should be added to **1**–**6** in such a way that the resulting derivatives can be stabilized not only in the active site but also in other peripheral binding pockets.

**Table 2:** Comparison of the inhibitory activities of 1–6 for PTPRQ and PTPRO

**Compounds**	**% inhibition at 100 μM for PTPRQ**	**% inhibition at 100 μM for PTPRO**
**1**	71.1 ± 0.8	94.4 ± 0.5
**2**	57.7 ± 0.4	94.2 ± 1.5
**3**	50.9 ± 2.2	48.7 ± 2.1
**4**	82.6 ± 0.2	95.0 ± 3.5
**5**	62.5 ± 1.6	95.0 ± 0.8
**6**	57.0 ± 2.1	81.2 ± 0.9

## Conclusions

In summary, we have identified six novel inhibitors of PTPRQ by applying a computer-aided drug design protocol involving the structure-based virtual screening with docking simulations under consideration of the effects of ligand solvation in the scoring function. These inhibitors are expected to have desirable physicochemical properties as a drug candidate and reveal a moderate activity with IC_50_ values ranging from 29.9 to 85.7 μM. Therefore, each of the newly discovered inhibitors deserves consideration for further development by SAR studies to optimize the antiobestic activities. The results of binding mode analysis with docking simulations indicate that the inhibitors can be stabilized in active site by the simultaneous establishment of multiple hydrogen bonds and van der Waals contacts.

## Competing interests

The authors declare that they have no competing interests.

## Authors’ contributions

HP: Developed methodology and wrote paper, KRU and BK: Carried out enzyme inhibition assays, BYK and SJK: Designed research plan and wrote paper. All authors read and approved the final manuscript.

## Supplementary Material

Additional file 1 Contains the structures of virtual hits without significant inhibitor potency for PTPRQ. Click here for file
