# Supplementary material for: Identification of novel PTPRQ phosphatase inhibitors based on the virtual screening with docking simulations
Source: Theor Biol Med Model. 2013 Aug 28;10:49. doi: 10.1186/1742-4682-10-49 (PMC3765866; doi:10.1186/1742-4682-10-49)
Supplement: Additional file 1 — Contains the structures of virtual hits without significant inhibitor potency for PTPRQ. [file 1742-4682-10-49-S1.doc]

**Structures of virtual hits without significant inhibitor potency for PTPRQ**
